# From engineered standards to natural bone: re-constraining topographical scanning for heritage samples

**DOI:** 10.1038/s41598-026-50016-0

**Published:** 2026-04-24

**Authors:** Christopher G. Tompkins, Holly Miller

**Affiliations:** 1 Department of Mechanical, Materials and Manufacturing Engineering, University of Nottingham, Nottingham, NG8 1BB United Kingdom; 2 Department of Classics and Archaeology, University of Nottingham, Nottingham, NG7 2RD United Kingdom; 3 Nottingham Materials and Environment Science and Heritage (N-MESH) network, Nottingham, United Kingdom

**Keywords:** Biological techniques, Health care, Medical research

## Abstract

The microscopic topographical scanning of bone material is a common practice for multiple fields, such as archaeology, medicine, and criminology. However, assessments of whether the tools used to perform these scans are accurate on these objects have yet to be performed. In this work, the performance of the two most common scanning techniques used on bone items, focus variation and 3D confocal scanning, are preliminarily assessed, revealing previously unknown limitations with current measurement practice and technologies. Across a range of bone specimens, the same limitation appears, which does not occur on any of a subset of engineered materials tested. The types of features that bone-focused work would commonly asses, such as cutmarks, are revealed to be the most susceptible to distortion. 3D confocal is preliminarily determined as the more accurate technique to use on bone. The discrepancies discovered in this work begin to answer some of the unsolved repeatability problems noted during previous work on bone objects, and highlight how future equipment must be redesigned to accommodate this new style of emerging, critical, applications.

## Introduction

Microscopic topographical imaging techniques, originally developed for industrial surface metrology, have begun to see wide use in other fields due to their forgiving use; they often produce true-colour and non-contact topographical (colloquially called 2.5D) scans of objects in a matter of seconds, in a regular lab environment and often without object preparation. Of these non-engineered use cases, heritage science^1,2^, medicine^3,4^, and criminology^5,6^ have particularly begun to adopt them, thanks to the detail they can non-invasively provide on an objects natural surface properties or incurred damage. Features on the surfaces of bone are key examples: Scanning ancient surface wear to determine if marks on bones were made post/pre/peri-mortem^7^, determining what materials bone tools were used on^2^, distinguishing between cutting actions^8^, and otherwise providing clear identification of small features to understand how a bone was treated^9^. Having evolved from engineering and industry, these measurement procedures already have rigorous protocols to ensure accuracy, repeatability, and certification compliance^10^. Although these studies have followed these measurement protocols without flaw^11^, researchers analysing bone items have highlighted replicability problems in their measurements (which industry and metrologists have not likewise encountered) that are confounding their conclusions^2^.

This replicability problem when collecting topographical scans of bone is further complicated by there being multiple imaging techniques, with the optical microscope methods of 3D confocal and focus variation^12^ generally being the most commonly used techniques in these studies^2,7,9,13–16^. Although similar in operation, so much so that some advanced equipment combines the techniques into a single device^17^, the operating principle behind both techniques differ: In 3D confocal microscopy, a high numerical aperture objective lens (or pinhole) is used to reject light that is not within a tight focal range (commonly sub-micron)^12^. By moving this objective perpendicular to the surface, and therefore also moving the focal plane relative to the object being scanned, the position of the object which backscatters light may be found by detecting peaks in intensity^12^. This can be repeated pixel-wise, for a camera viewing the surface through the objective, to reconstruct an areal height-map of the surface. In the focus variation technique, out-of-focus light is not rejected, and an areal image is always captured^12^. Local fluctuations in contrast in the image, between neighbouring pixels, is then measured as the objective is moved perpendicular to the surface. The surface is considered to be at the focal point of the objective when the contrast is highest, once again allowing an areal height-map to be constructed^12^.

Due to the different operating principles, both of these techniques are known to be best-suited to different measurement scenarios, performing best on different materials, or even the same material with different physical surface properties^12^. For example, when structured in a different way, such as polished vs 3D printed steel, the different surface roughnesses have been shown to dramatically affect the quality of a scan^18^. As well as physical properties, optical properties such as colour will naturally affect these optical methods differently. This susceptibility to the physical and optical characteristics of a surface means that scanning a new material type can introduce unforeseen errors and therefore needs to be assessed for suitability, something which has yet to occur with bone samples, to the authors’ knowledge.

This is a problem which has yet to cycle back to measurement science, likely due to how infrequently the required fields interact, and where this work continues from. A number of challenges hinder solving this knowledge-gap, the primary one being that bone is a complex natural material^19^ which cannot be manufactured into the precise measurement artefacts needed to perform the complete analyses without altering the surface properties (e.g. changing the roughness or causing burning through milling)^3^. Instead this work focuses on a preliminary step: *what measurement discrepancies occur when common scanning technologies are used on bone, and are these discrepancies unobserved in the engineered materials that can (and are) made into measurement artefacts?* From this analysis, the two common scanning techniques that are used on bone are compared, the best-suited method proposed, and the type of surface features which they best capture are determined.

## Methodology

### Ethics declaration

All bones used in this work come from legacy teaching & research collections, recovered as surface finds or other otherwise already decontextualised, and held at the University of Nottingham department of Classics and Archaeology. All samples originate from the UK and have not been imported. No external ethical approval was required, and no formal permission was required as the analysis is non-destructive. No animals were harmed for the purposes of this research.

### Equipment

One device, a Sensofar S-Neox Optical Profilometer^17^, was used to perform the topographical analysis in this work. This device is capable of scanning using both focus variation and 3D confocal techniques, so no other device was needed. Crucially, the profilometer can measure using either technique without any hardware changes or movement, meaning the same microscopic area of surface can be imaged down to a minimum manufacturer-determined accuracy of 1*nm*.

**Fig. 1:**
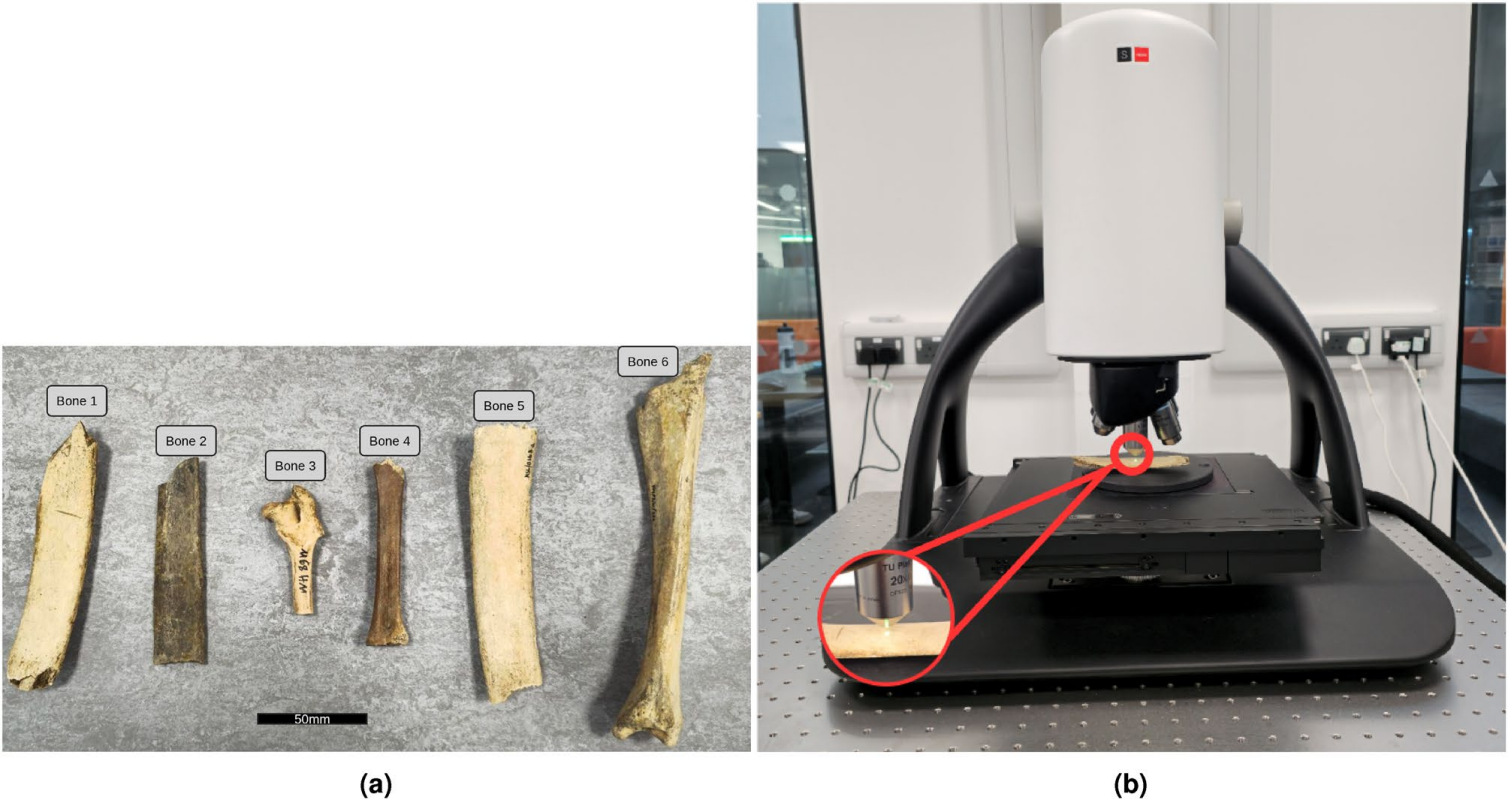
In (**a**), the six archaeological bone samples analysed in this work can be seen. Labelled 1 through 6, each bone is (respectively) a: medial rib section of a large mammal, medial rib section of a large mammal, proximal hare (Lepus sp.) femur, proximal sheep (Ovis sp.) metacarpal, medial rib section of a large mammal, and distal pig (Sus sp.) tibia. In (**b**), one of the samples can be seen mounted in the topographical scanner. All work was performed in a controlled environment, and all bones were allowed to rest there for a minimum of 48 hours before they were scanned.

Six bone samples were chosen for the analysis, show in Fig. 1 below, each an archaeological artefact but with unique properties:

1. Medial rib section from a large mammal.
2. Medial rib section from a large mammal.
3. Proximal hare (Lepus sp.) femur.
4. Proximal sheep (Ovis sp.) metacarpal.
5. Medial rib section of a large mammal.
6. Distal pig (Sus sp.) tibia.

In this context, a large mammal is defined as a horse, cow, or red dear. As ribs are not diagnostic, further classification is not possible (without ancient-DNA analysis or similar, potentially^20^). Each bone also comes from different recovery environments or burial contexts, and therefore have experienced unique surface alterations from chemical and thermal changes (such as cooking or acidic soil), which is also known to vary the surface and optical properties of bone^21,22^. The varying discolouration is one visible indicator of these unknown processes. Each bone therefore had a different surface roughness and mineral composition^23^, respectively changing their topography and optical properties. Next to the samples in Fig. 1, an example of one of the bones on the profilometer has been included, to show the scanning set-up. Each sample was allowed to rest in the (temperature and humidity controlled) lab environment for a minimum of 48 hours before it was scanned, to allow it to reach thermal equilibrium.

To compare the results gathered from bone to the results expected during the development of topographical imaging standards^10^ and equipment design, samples of engineered materials were also sourced. The engineered materials used were silicone carbide, stamped polished brass, and 3D printed steel. All samples were scanned at $\times 20$ magnification, using focus variation and 3D confocal techniques back-to-back.

### Residuals between techniques

The initial stage of this work compared the two imaging methods on a single piece of bone at an identical areal position. The differences between the two hight maps was then analysed, by plotting the residuals as a histogram. These differences then form a statistical distribution. If all sources of error are truly random, as should be the case for the calibrated and certified device (and later shown with the engineered materials), this distribution should be symmetric, and begin to be skewed if not^24^. In a general form, the shape of the statistical distribution these data should take will be a skew-normal^24^ distribution *f*(*x*) :

1 $$\begin{aligned} f(x) = \frac{2}{\Omega } \phi (\frac{x - \xi }{\Omega })\Phi (\frac{\alpha (x-\xi )}{\Omega }), \end{aligned}$$

where $\xi$ determines where the peak of the distribution is centred, $\Omega$ determines its width, and $\alpha$ governs the asymmetry. $\phi$ is a Gaussian probability density function, and $\Phi$ is the associated Gaussian cumulative distribution function^24^.

Depending on the exact shape of this curve, there are multiple possible ways to interpret the source of the differences between the two imaging techniques:

- **Gaussian centred at 0 (**$\xi$ == 0, $\alpha$ == 0**)**: the two methods are equally accurate and precise, only random noise drives uncertainty. In real-world experiments, $\xi$ will be slightly larger than zero, due to measurement accuracy ($\pm 10nm$ in these experiments, from the equipment manufacturers data-sheet).
- **Gaussian with an offset centre (**$\xi$!= 0, $\alpha$ == 0**)**: the two methods are equally accurate and precise, but there is a systematic error between the two methods (e.g. misalignment).
- **Skew-normal distribution (**$\xi$ == $\mathbb {R}$, $\alpha$!= 0**)**: the two methods are not the same; one or both of the measurement methods is measuring an additional non-random and non-systematic deviation.

### Normality threshold testing

This process of binning data into a statistical distribution was then repeated for every bone and engineered sample, and the skewness ($S_k$) of each distribution calculated, which could be completed automatically using MATLAB, using the standard expression of skewness^25^:.

2 $$\begin{aligned} \text {Sk} = \frac{4-\pi }{2} \cdot \frac{\delta ^3}{\left( 1 - \frac{2\delta ^2}{\pi }\right) ^{3/2}}, \quad \text {where } \delta = \frac{\alpha }{\sqrt{1+\alpha ^2}}. \end{aligned}$$

The two-dimensional surface roughness (*Sa*) of each sample was also calculated for each, following ISO 25178^10^. The skewness of every sample was then plotted against the surface roughness, and any relation examined, particularity noting which sample types had non-Gaussian skewness.

Although a true Gaussian distribution will have value of $|S_k| == 0$, real data will always be a sub-sample of this continuous distribution and therefore even a Gaussian distribution will have a measurable non-zero skewness, but within a finite range dependent on the quantisation and algorithm used^26^. Any value outside of this range will indicate a non-Gaussian distribution^25^. To locate the transition point between Gaussian and non-Gaussian regimes, 20,000 synthetic Gaussian data sets were simulated, each matching the experimental data in both resolution and sample count (which was consisted throughout). This was achieved using the inbuilt Gaussian random number generator in MATLAB. The skewness of each distribution was then found, and the maximum value of absolute skewness ($|S_k|$) from the 20,000 possible values was taken as the transition point. 200,000 datasets were chosen to establish a statistically robust $>4.5\sigma$ confidence level^27^ in the skewness threshold, under a worst-case assumption where the 200,001st value falls outside of the observed range set by the first 200,000.

### Variance within images

To give an indication of which method had higher distortion when imaging bone, a variance between first differences approach was employed^28^. The first differences of a dataset of values *y*, is simply the difference between successive values:

3 $$\begin{aligned} \Delta y_i = y_i - y_{i-1}, (i = 1,2,3,...). \end{aligned}$$

The variance of these changes captures how steadily the dataset changes from one value to the next, and can be expressed as

4 $$\begin{aligned} Var(\Delta y_i) = \frac{1}{n-1} \sum _{i}^{n} \left( (y_i - y_{i-1}) - \overline{\Delta y} \right) ^2, \end{aligned}$$

where *n* is the total number of values in the data and $\overline{\Delta y}$ is the data mean. No matter their type, from systematic to random, sources of measurement error will increase this value. If there are multiple datasets measuring the same feature, then the most unstable dataset, the one with the highest $Var(\Delta y_i)$, is therefore likely to have the highest deviation from the true value. This assertion is based on the fact that variance of first differences measures pixel-to-pixel changes relative to their mean value, capturing local gradient fluctuations. Given that the surface features are at least an order of magnitude larger than the pixel scale ($\sim 100nm$)^17^, both in this work and in literature^19^, the true surface can be assumed to have a continuous gradient at the measurement resolution. Consequently, pixel-to-pixel fluctuations should tend toward zero in an ideal setting (or a minimum baseline in the presence of measurement noise), meaning that excessive fluctuations in the data are attributable to measurement deviation rather than genuine topographical variation. However, as real changes in surface topography would increase the value of $Var(\Delta y_i)$ along with measurement error, it is not possible to directly compare two different surface areas. Instead, only the relative differences between identical areas of individual samples were investigated.

## Results

**Fig. 2:**
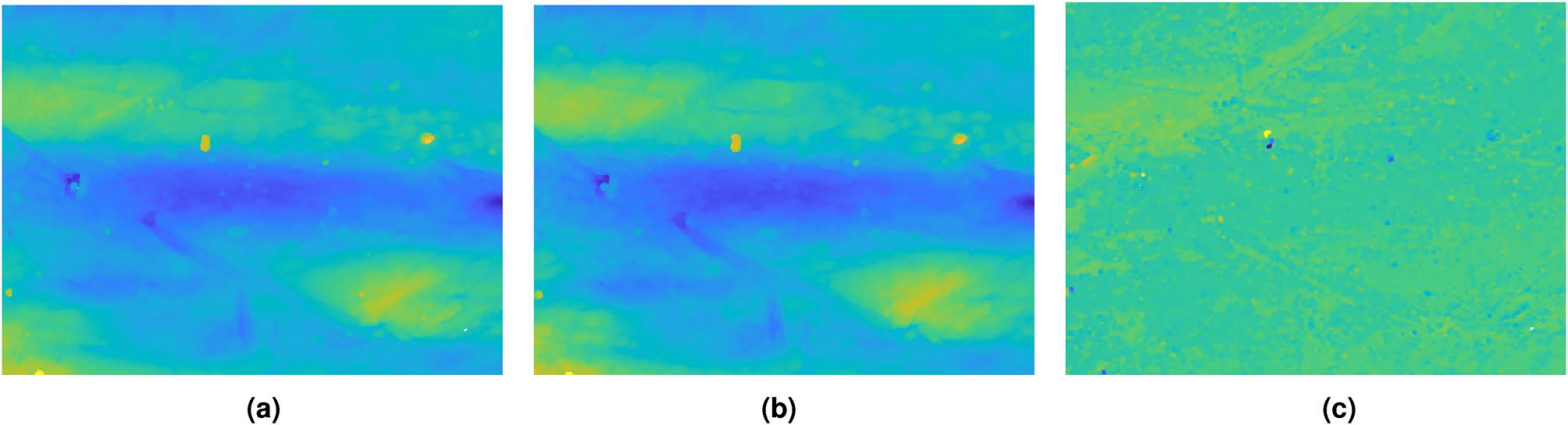
Two examples height map images of a bone surface, taken using both 3D confocal (**a**) and focus variation (**b**) techniques. Each image is of an identical area of the surface, and was taken at $\times 20$ magnification using a Sensofar S-Neox Optical Profilometer. By eye, each image initially looks identical (differences can be seen on closer inspection). Further investigation reveals non-visible distortions between the two which affect measurements, illustrated in (**c**) by subtracting the confocal data from the focus variation data, which also successively affects results and values based on these measurements.

An initial, visual-only, comparison which shows how scans appear, can be seen in Fig. 2 below. One scan has been taken with focus variation, and the other using the 3D confocal technique, both at the same arbitrary position on Bone 1. Differences between the two scanning techniques can be seen by eye, however they are subtle and can be easily missed on a cursory glance.

These differences become more noticeable when the images are subtracted from each other and the residuals are inspected.

### Residuals between techniques

**Fig. 3:**
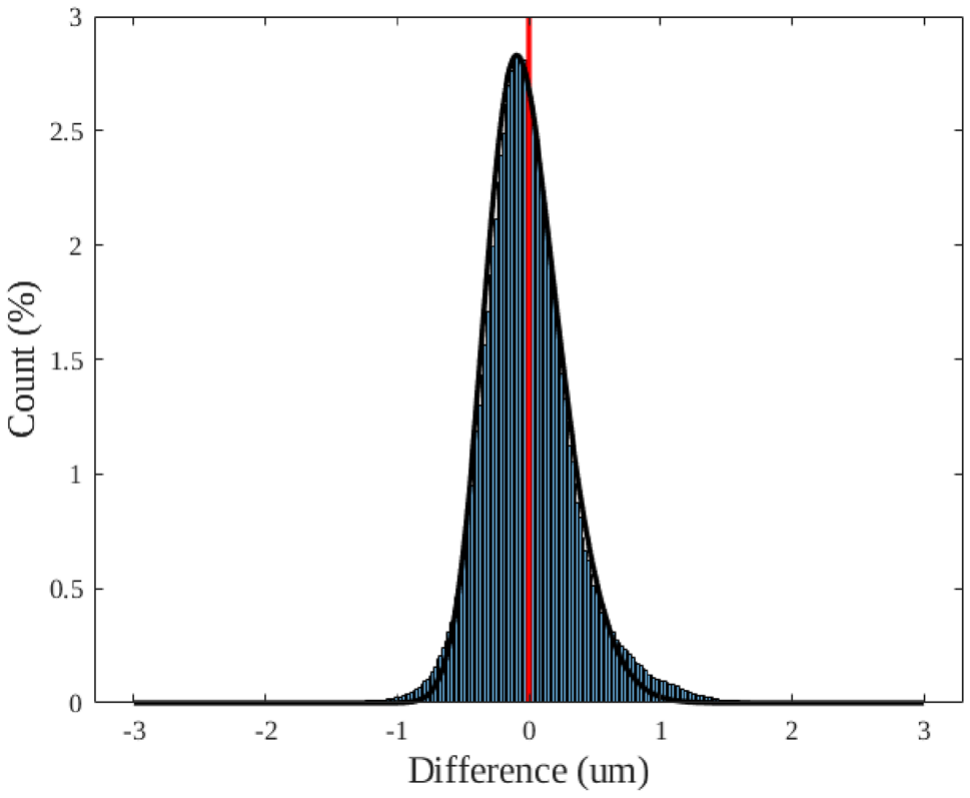
The binned residuals between height-maps taken using the 3D confocal and focus variation techniques, when imaging an identical area of a bones surface, using a Sensofar S-Neox Optical Profilometer. Assuming only truly random fluctuations, such as sensor shot noise, then this distribution should be Gaussian with a centre of $\pm 10nm$ (indicated by the red vertical bar, which starts and stops at the limits of this range, having a total thickness of 20*nm*). This is not the case, and through fitting a skew-normal distribution to determine if the distribution is Gaussian and its true centre, a skewed distribution with a centre of $-0.31 (\pm 0.01) \mu m$ was found to fit to the distribution (indicated by the black line). This is indicative of a non-random and non-systematic error between the measurement techniques. However, the sample mean of the distribution does fit within this $\pm 10nm$ range, at 3*nm*.

An example of the binned residuals, the differences between the 3D confocal and focus variation height-maps, can be found in Fig. 3 below. This example continues to use Bone 1. The expected centre of the distribution, the uncertainty between the two measurement techniques given by the device manufacturer, is indicated as a red vertical bar, showing that the peak of the distribution does not intersect. By using Equation 1 to fit a skew-normal distribution to the data (the fit being the black overlay in the figure), it can be seen that this is not due to an offset in the data, but skewness.

The fit parameters were found to be $\xi$ = −0.31 $(\pm 0.01) \mu$m, $\omega$ = 0.42 $(\pm 0.01)\mu$m, and $\alpha$ = 1.95 $(\pm 0.02)\mu$m. Uncertainties on the parameters come from the 95% confidence intervals of the fit. This follows the trend outlined by case 3 in the method section. It is also important to note that, although there is a skewness present, the sample mean is still within the expected range from the manufacturer even if the peak is not: $- 3nm$ vs $\pm 10nm$, respectively, with a peak value of $-310 nm$. Therefore, averaged values will not be distorted from the true value (e.g. mean surface roughness), but single-measurement values will be susceptible (e.g. cutmark depth).

To see whether this skew-inducing distortion held true for other samples, this was repeated (for bones and engineered samples), and the skewness of the distributions collected together.

### Normality threshold testing

**Fig. 4:**
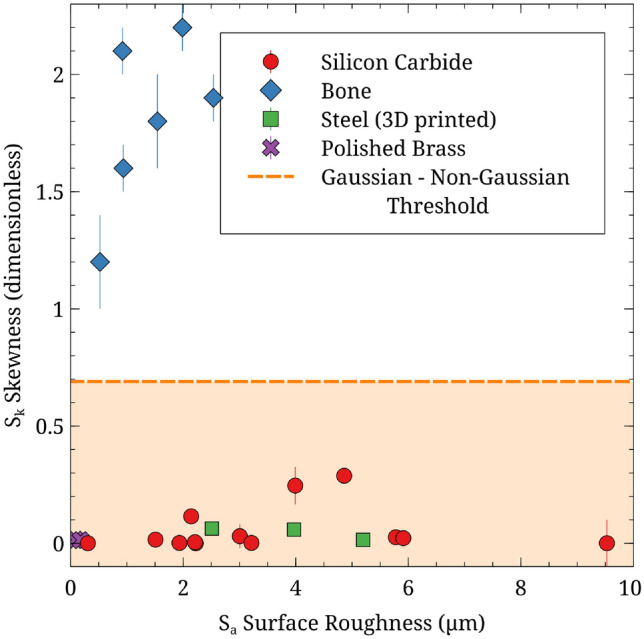
The Skewness ($S_k$) of residual-histograms plotted as a function of surface roughness ($S_a$) of a set of samples. The residuals are the point-wise differences between 3D confocal and focus variation images, taken of identical sample areas. Multiple engineered materials (silicon carbide, steel, and brass) are compared against bone. Based on sampling 20,000 true-Gaussian datasets, values of $S_k$ above 0.69 indicate a sample has a non-Gaussian distribution and therefore the differences between sample images cannot be explained by random noise. All engineered samples have $S_k<0.69$ (actually greater than an order of magnitude less, averaging 0.05), and all bone samples have $S_k> 0.69$. No correlation between surface roughness and skewness is observed. All values have uncertainites, taken from the 95% confidence intervals on the skewness fits, although most are too small to plot.

The collective skewness values can be found in Fig. 4. Through the 20,000 simulated distributions outlined in the methods section, it was determined that a sub-sampled Gaussian distribution would have a skewness of 0.69 or less. This has been indicated on the graph in orange, with the transition region as a horizontal (orange) dashed line. These skewness values have been plotted as a function of the surface roughness, with their uncertainties coming from the 95% confidence intervals of the skewness fits. Of all engineered and non-engineered samples tested (bone, silicon carbide, steel, and brass), only the bone samples have a skewness above the Gaussian threshold; this effect is not seen in any tested engineered material which instead have an average skewness greater than an order of magnitude less than the transition threshold (0.05 vs 0.69, respectively).

It can therefore be seen that every bone sample has a skewness which indicates there is a non-random and non-systematic difference between the two topographical scanning methods (case 3 in section 3.3), which is being driven by a property present in every bone but not the other tested materials, seemingly exclusive of its type or chemical treatment. No dependence on surface roughness alone can be determined from the set of samples, and therefore another property must be contributing to the distortions. This is unsurprising, as (at microscopic length scales) bone is a complex matrix of crystals (rather than a monocrystal, such as the silicon carbide samples) and other materials that have varying and non-linear optical properties^19^. The main crystal in bone, hydroxyapatite, is known to be birefringent and stress can be frozen in to these crystals along the growth direction^29^, for example.

To give an indication of which method is likely the most accurate for scanning bone, the variance of first differences from individual bone scans were analysed next.

### Variance within images

**Fig. 5:**
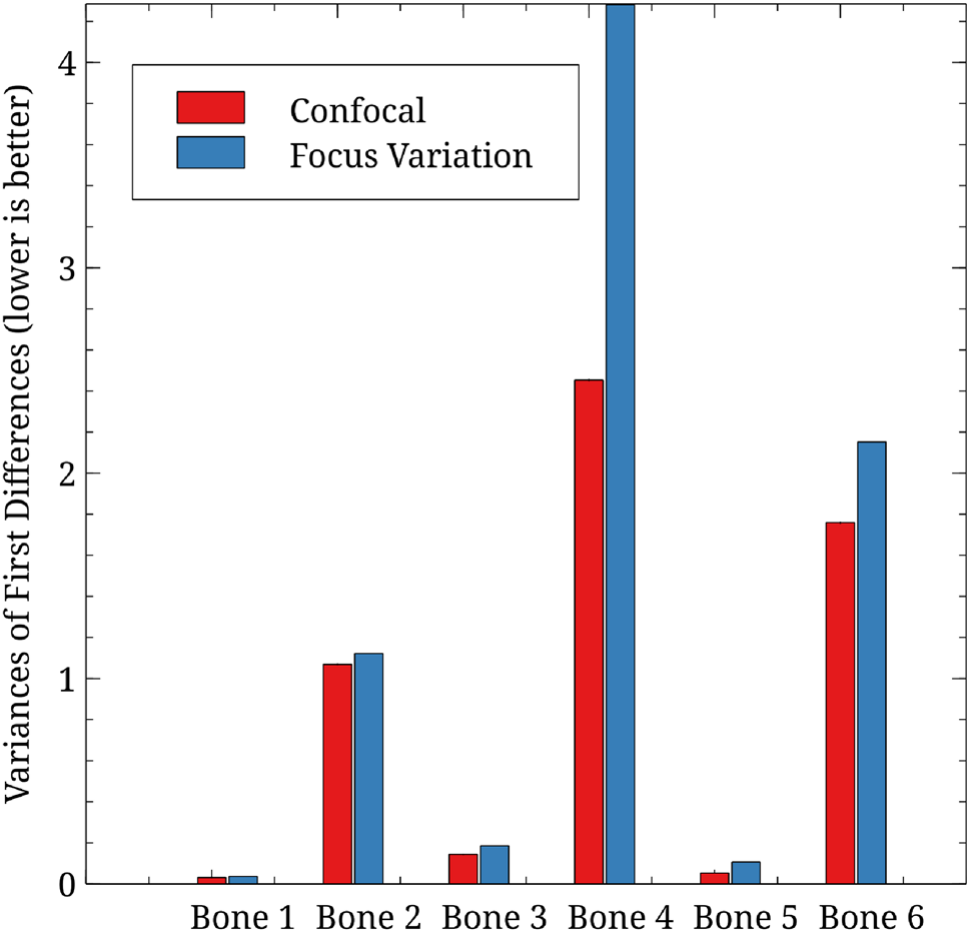
The variance of first differences, the changes in topographical height from one position to the next, from 6 bone samples measured using 3D confocal and focus variation. 3D confocal data is in red, and focus variation is in blue. On every sample, focus variation has the highest variance and therefore does not evolve as smoothly from one point to the next. This higher instability implies that this method has higher measurement error, when used on bone. Uncertainties are calculated as the standard error of the variance and are too small to plot.

The values of $Var(\Delta y_i)$ found using Equation 4 for all six bone samples are shown in Fig. 5 below, grouped such that 3D confocal and focus variation techniques are compared for each individual sample only. Although the variance fluctuates from sample to sample, these measurements show a consistently larger fluctuation between neighbouring points in focus variation images, compared to confocal images. This consistently lower noise level in confocal scans implies that this technique is the preferable method for topographically scanning bone.

### Overall interpretation

It is clear from these results that there is some physical process occurring during confocal/focus variation-based topographical scans of bone that does not occur when common engineered samples are scanned. This unaccounted-for process affects the accuracy of the scans in a way which does not affect statistical measurements (e.g. surface roughness), evidenced by there being no offset in the sample means of measured statistical distributions. Since statistical measurements aggregate multiple single values into a distribution that suppresses individual uncertainties, which may be across the entire range of the distribution, single measurements (e.g. cutmark depths) bear the full effect of any measurement distortion. As these single-value-type measurements are commonly used in multiple fields to analyse damage to bone (including at scales where the added uncertainties seen here would be impactful^7,15,16,21^), where accuracy may impact human health or freedom related decisions, ensuring that this limitation is understood and accounted for is critical.

These results indicate that the 3D confocal technique is likely the most accurate of the two scanning methods, although whether both methods have distortion cannot be concluded. The source of these imaging distortions has been found to not correlate to the bones surface roughness, and is likely a complex phenomenon in need of a larger dataset. To conclusively resolve these remaining questions, ISO-compliment areal measurement artefacts that capture the physical and optical properties of bone will be needed^10^. The development must be thorough, accounting for the full range of bone compositions and the chemical processes that modify them. This will ensure that necessary hardware and/or software adjustments to the measurement devices will comprehensively cover every possible measurement scenario. Although this technology limitation has so far only been found in bone samples, it is reasonable to assume that other materials cause similar discrepancies which have also yet to be observed. Determining the physics behind these distortions, so a collective group of affected materials can be identified, will be necessary to understand and overcome the limits of the current technologies.

## Conclusion

This preliminary work has found unaccounted-for non-random and non-systematic discrepancies between the two common topographical imaging technologies used on bone samples, determining that they are most suited to measuring statistical surface properties of bone rather than single-measurement features. This has particular implications for archaeological and criminological applications, where single-measurement defects on bone (such as cut marks) may be distorted in shape without visual cues to be detected by humans. As this has the potential to affect scientific study and human lives directly, ensuring these measurements are accurate is critical. Based on this work, it is currently recommended that the 3D confocal technique is used over focus variation, as this appears least susceptible to the distortions introduced by the sample. The exact cause of these discrepancies, and whether they are present in other types of samples, is not yet known, although to date they do not appear in any tested engineered samples. This warrants further investigation, to determine what physical or optical property is not being accounted for, and the development of ISO-compliant bone-analogue measurement artefacts to ensure suitable tool development in the future.

## Data Availability

The datasets generated during and/or analysed during the current study are available in the Zenodo repository, 10.5281/zenodo.19697930.

## List of symbols

${S_k}$: Skewness of a distribution.
${S_a}$: Two-dimensional surface roughness.
*f*(*x*): Skew-normal distribution.
${\xi }$: where the peak is centred.
${\Omega }$: governs how wide the distribution is.
${\alpha }$: adjusts the asymmetry.
${\phi }$: Gaussian probability density function.
${\Phi }$: Gaussian cumulative distribution function.
${Var(\Delta y_i)}$: Variance of first differences, between elements $y_i$.
${\overline{\Delta y}}$: Sample mean, of elements $y_i$.
